# Effects of More-Affected vs. Less-Affected Motor Cortex tDCS in Parkinson’s Disease

**DOI:** 10.3389/fnhum.2017.00309

**Published:** 2017-06-12

**Authors:** Giuseppe Cosentino, Francesca Valentino, Massimiliano Todisco, Enrico Alfonsi, Rosaria Davì, Giovanni Savettieri, Brigida Fierro, Marco D’Amelio, Filippo Brighina

**Affiliations:** ^1^Department of Experimental Biomedicine and Clinical Neurosciences (BioNeC), University of PalermoPalermo, Italy; ^2^Department of Neurophysiopathology, C. Mondino National Institute of Neurology Foundation, Istituto di Ricovero e Cura a Carattere Scientifico (IRCCS)Pavia, Italy

**Keywords:** non-invasive brain stimulation, tDCS, cortical excitability, motor cortex, Parkinson’s disease

## Abstract

**Objective**: To evaluate therapeutic potential of different montages of transcranial direct current stimulation (tDCS) in Parkinson’s Disease (PD) patients with asymmetric motor symptoms.

**Materials and Methods**: Fourteen patients with asymmetric PD underwent, while on treatment, seven separate sessions including electrophysiological and clinical evaluation at baseline and after anodal, cathodal and sham tDCS of the primary motor cortex (M1) of the two hemispheres. Changes in motor cortical excitability were evaluated by transcranial magnetic stimulation (TMS). Effects on motor symptoms were assessed by testing finger tapping (FT) and upper limb bradykinesia, and by using the Italian validated Movement Disorder Society revision of the Unified PD Rating Scale (MDS-UPDRS).

**Results**: Only anodal tDCS of the more-affected M1 (contralateral to the more-affected body side) and cathodal tDCS of the less-affected M1 (contralateral to the less-affected body side) were able to induce significant changes in cortical excitability, i.e., facilitation and inhibition of the motor evoked potentials respectively. The motor performances of both hands significantly improved after anodal tDCS of the more-affected M1, as well as after cathodal tDCS of the less-affected one.

**Conclusion**: Our findings support the potential usefulness of tDCS as add-on treatment for asymmetric PD, also providing interesting clues on the possible pathophysiological role played by an asymmetric activation of homologous motor cortical areas in PD.

## Introduction

In recent years, non-invasive brain stimulation (NIBS) techniques have been increasingly used, with therapeutic purposes, in different pathological conditions characterized by an altered state of cortical excitability (Rossini et al., 2015). Among NIBS techniques, transcranial direct current stimulation (tDCS) has become one of the most widely used, due to easy to use and useful for sham-controlled double-blind experiments (Priori, 2003). A few studies have shown that tDCS, when applied to the motor cortex, is safe and may induce clinical improvement in patients with Parkinson’s disease (PD; Fregni et al., 2006; Benninger et al., 2010; Valentino et al., 2014). The rationale of using tDCS for treatment of PD is based on experimental findings of abnormal motor cortical activation, that has been considered as a neurophysiological correlate of the extrapyramidal motor symptoms (Cantello et al., 1991; Chen et al., 2001; Lefaucheur et al., 2004). In particular, neuroimaging (Sabatini et al., 2000; Haslinger et al., 2001) and neurophysiological (Cantello et al., 1991; Valls-Solé et al., 1994) studies have provided evidence of bilateral asymmetric increase in motor cortical excitability. The pathophysiological meaning of motor cortical hyperactivation, however, still remains debated, as it could either represent an adaptive motor strategy attempting to compensate for the reduced thalamocortical drive, or be expression of maladaptive phenomena (Ridding et al., 1995; Sabatini et al., 2000; Pierantozzi et al., 2001; Thobois et al., 2004; Wu et al., 2007). Furthermore, theoretically, the pathophysiological role of motor cortical hyperexcitability could be different in the two hemispheres in patients with asymmetric motor impairment. In recent years, different neurophysiological studies have provided evidence of both asymmetry in primary motor cortex (M1) excitability (Wu et al., 2007; Kojovic et al., 2012) and reduced transcallosal inhibition from the more-affected hemisphere (i.e., contralateral to the more-affected side of the body) to the less-affected one (Li et al., 2007; Spagnolo et al., 2013). Both of these may be consequence of the asymmetric impairment of the striato-frontal motor circuit, and they have been supposed to contribute to the asymmetric motor impairment that characterizes the disease.

Though the antiparkinsonian medication may reduce between-hemispheres differences in cortico-striatal activity, the persistence of clinical asymmetry in patients on medication suggests that a physiological interhemispheric balance could not be achieved (Spagnolo et al., 2013). In this view, the use of NIBS techniques could be useful thanks to their ability to exert neuromodulatory effects on targeted cortico-subcortical neural networks of one hemisphere.

On these bases, the aim of this work was to evaluate whether the effects of tDCS on clinical symptoms might be different when targeting the M1 of the more- or the less-affected hemisphere in PD patients with asymmetric motor impairment. The working hypothesis was that if a functional interhemispheric imbalance contributed to the clinical motor deficits, then either the “activation” of the more-affected hemisphere by anodal tDCS, or the “inhibition” of the less-affected one by cathodal tDCS, would result in a clinical benefit. Although it is usually thought that anodal tDCS increases cortical excitability and cathodal tDCS reduces it (Nitsche and Paulus, 2000; Nitsche et al., 2003a), there is evidence that the effects on cortical excitability may significantly vary in relation to the stimulation parameters used (Batsikadze et al., 2013) and may be different in pathological conditions characterized by altered cortical excitability (Nitsche et al., 2008). Very little is known about the effects of tDCS on motor cortical excitability in PD (Fregni et al., 2006). This is why we decided to apply both anodal and cathodal tDCS as well as sham (placebo) tDCS to the M1 of the two hemispheres and to evaluate, along with modifications in clinical measures, also changes in motor cortical excitability by means of transcranial magnetic stimulation (TMS).

## Subjects and Methods

### Subjects

Sixteen patients (8M/8F, aged 58 ± 11.5 years) with idiopathic PD diagnosed according to the U.K. PD Brain Bank criteria (Hughes et al., 1992) were enrolled in the study. Only PD patients with asymmetric motor symptoms as assessed by lateralized Movement Disorder Society revision of the Unified PD Rating Scale (MDS-UPDRS) scores during the on-state were recruited. A minimum 2-point difference between the two sides of the body was considered clinically significant. Throughout the manuscript, we will refer to the more-affected hemisphere as that contralateral to the side of the body with the lower lateralized (MDS-UPDRS) score.

All patients enrolled were required to be on a stable dose of antiparkinsonian medications for at least 2 months (mean levodopa equivalent dose of 386.2 ± 233.5 mg calculated according to Tomlinson et al., 2010), and were expected to require no medication adjustments during the course of the study. Only patients with fairly stable disease as assessed by a clinical evaluation carried out twice at a 2-month interval (no total MDS-UPDRS score changes ≥2 between the two evaluations) were enrolled.

All patients had a score at the Mini Mental State Examination ≥26. Exclusion criteria were previous treatment with deep brain stimulation, history of seizure disorder or stroke and psychiatric illness.

Before enrollment, all subjects were checked for contraindications to NIBS (Keel et al., 2001; Poreisz et al., 2007). The clinical data of the patients are summarized in Supplementary Table S1. The study was approved by the local ethics committee of the University Polyclinic of Palermo, and written informed consent to participate was obtained from all subjects prior to the experiment according to the Declaration of Helsinki.

### Study Design

**Figure 1:**
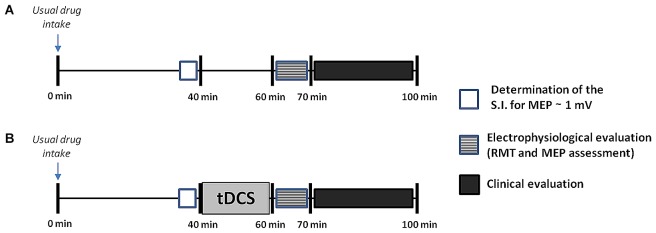
Design of the study. After inclusion, patients underwent seven separate sessions including a baseline session **(A)**, in which no transcranial direct current stimulation (tDCS) was applied, and six tDCS sessions **(B)**, in which anodal, cathodal or sham tDCS was applied for 20 min to the primary motor cortex (M1) of the more- or the less-affected cerebral hemisphere. All sessions were performed in a randomized order at least 3 days apart. Both in the baseline and in the tDCS sessions the electrophysiological assessment and the clinical evaluation were carried out at the same time interval from the last drug intake, i.e., after 60 and 70 min respectively. The electrophysiological evaluation took about 10 min to complete, whilst the clinical assessment took another 30 min or so. This latter included, always in the same order, assessment of the finger tapping (FT) test, test of upper limb bradykinesia, and Movement Disorder Society revision of the Unified PD Rating Scale (MDS-UPDRS). For more details see explanation within the text. SI, stimulation intensity.

**Figure 2:**
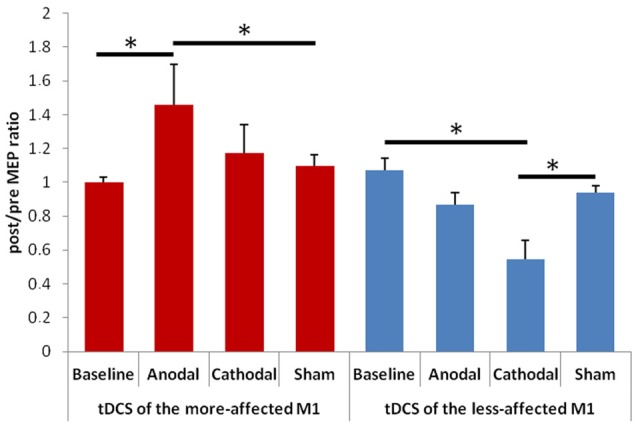
Changes in amplitude of the motor evoked potentials (MEPs) elicited by transcranial magnetic stimulation (TMS) of the more- (on the left) and less-affected (on the right) M1. Error bars indicate standard error of means (SE). Horizontal bars indicate significant differences (*p < 0.05).

The study followed a double-blind, randomized, crossover and sham-controlled design. A physician different from that who applied the tDCS interventions completed the clinical and electrophysiological evaluations. Both patients and evaluators were unaware of the type of stimulation (active or sham-tDCS) performed in each experimental session. Patients were evaluated during the “on” state in seven separate sessions performed on different days, at least 3 days apart (Nitsche et al., 2008). In each patient, all the experimental procedures were carried out at the same time of the day, to avoid possible circadian influences and to assure that the “on” state was comparable among sessions. The different sessions were conducted in a randomized order using a random sequence generator, and included: (1) a baseline session, in which the electrophysiological and clinical assessment was performed in the absence of any stimulation intervention, about 60 min after the patients took their usual doses of medication (Figure 1); (2) six tDCS sessions (three sessions for each hemisphere: anodal, cathodal and sham tDCS) in which the electrophysiological and clinical assessment was performed immediately after the end of tDCS (Figure 1). tDCS was always applied 40 min after the usual medication intake for 20 min, to allow the clinical and electrophysiological evaluation to be performed at the same time interval from drug ingestion as in the baseline session. This allowed us to avoid influence of the time interval from the last assumption of the antiparkinsonian medication on the outcome measures (Thirugnanasambandam et al., 2011).

### tDCS Intervention

tDCS was delivered through a pair of electrodes in a 5 × 5 cm saline-soaked synthetic sponge using a battery-driven constant current stimulator (BrainSTIM Transcranial Stimulator, Italy) and following safety guidelines (Poreisz et al., 2007). Currents were given for 20 min at a 2 mA intensity in patients at rest, and were ramped up or down over the first and last 30 s of stimulation. For the anodal stimulation condition, the anode was positioned over the M1 hotspot of the abductor pollicis brevis (APB) muscle at rest, while the cathode was placed over the contralateral orbitofrontal cortex. The reverse montage was used for cathodal tDCS.

We chose to apply anodal tDCS for 20 min at 2 mA intensity based on previous works (Benninger et al., 2010; Valentino et al., 2014) and on evidence that stimulation intensities higher than 1 mA may be more beneficial in enhancing performance in PD (Boggio et al., 2006; Broeder et al., 2015). The same stimulation parameters were used for cathodal tDCS both to avoid possible confounding factors when interpreting the results, and based on finding that the inhibitory effect of cathodal tDCS, when given at a lower intensity of 1 mA, may be less pronounced in patients with PD (Fregni et al., 2006).

For the sham condition, the intensity was set to 2 mA as for real tDCS, but the DC stimulator was only switched on for 30 s at the beginning of the sham session and then turned off. Thus patients felt the initial itching sensation, as for active-tDCS, but received no current for the rest of the stimulation period. At the end of the sham condition, the DC stimulator was switched on for 30 s to mimic the sensation of the ramp-down current perceived at the end of the active-tDCS. This technique has been reported as a reliable method of sham stimulation (Nitsche et al., 2008). All subjects underwent the experimental interventions when on-therapy to evaluate the therapeutic potential of tDCS as adjunctive treatment for PD.

### Electrophysiological Assessment

All patients underwent the electrophysiological assessment by means of TMS to evaluate the effect of tDCS on motor cortical excitability. TMS was performed using monophasic magnetic pulses with posterior–anterior (PA) current. MEPs were recorded from the APB muscle contralateral to the side of stimulation, by using 0.9-cm diameter Ag-AgCl surface electrodes placed 3 cm apart over the belly and tendon of the muscle. About 60 min after drug intake, we recorded in each session (baseline and post-tDCS): (1) the resting motor threshold (RMT), defined as the minimum intensity of stimulation needed to produce responses of 50 μV in at least 50% of 10 trials, and (2) the peak-to-peak amplitude of ten MEPs recorded in patients at rest with inter-stimulus intervals of 10 s. Based on the evidence that MEP amplitude is characterized by a high intraindividual variability over time (Wiethoff et al., 2014; Horvath et al., 2015), we used a pre/post test design to evaluate changes in MEP amplitude. Thus in each session (baseline, active and sham sessions), 35 min after the ingestion of the antiparkinsonian therapy, we assessed the stimulus intensity to obtain MEPs with an average peak-to-peak amplitude of around 1 mV and then we recorded a block of 10 MEPs (pre-MEPs). Afterwards, this adjusted stimulus intensity was used to record a block of 10 MEPs at 60 min after drug intake (post-MEPs) both in the baseline and after the end of tDCS (Figure 1). Post/pre MEP ratios were computed for each session and used for the statistical analyses. In this way, we controlled for the effect of medication intake on cortical excitability, but also ensured a control for spontaneous changes in cortical excitability.

Focal TMS was applied over the hand motor cortex by using a double-circular-70-mm coil powered by a Magstim stimulator (Magstim 200, Magstim Co., Dyfed, UK). The stimulating coil was placed over the optimal site for eliciting responses in the contralateral target muscle. The EMG activity was recorded with a bandpass of 10–1000 Hz at a sampling rate of 2 kHz, with a display gain ranging from 50 μV/cm to 1000 μV/cm. EMG signals were collected, averaged and analyzed off-line. Only EMG traces displacing no background EMG activity were used for the analysis, whilst EMG traces contaminated by voluntary or tremor-related muscle activity were discharged to avoid possible influences on MEP amplitudes. Throughout the period of the examination the subjects were given audiovisual feedback of EMG activity to help maintain muscle relaxation. TMS was performed following safety guidelines (Rossi et al., 2009).

### Clinical Evaluation

Lateralized movements of the upper limbs were evaluated by using two different motor tests. The finger tapping (FT) test was performed, with each hand separately, to evaluate rhythmic movements patterns according to a previously described method consisting of 50 FT cycles (Arias et al., 2012). Patients were asked to tap at their preferred rate (comfort) with the index finger. We used FT at “comfort” mode to avoid that appearance of fatigue, as it may be observed at “fast” mode, could be responsible for a drop in tapping rate (Aoki et al., 2003). In each trial, three initial discarded taps were performed to reach a steady state. All patients were videotaped during the execution of the FT test. The videos were rated offline by two different experts who calculated the mean FT rate.

Bradykinesia of hand and arm movements was assessed, on the two sides of the body, by recording the total time to perform the following sequence 10 times: (1) hand closing and opening; (2) elbow flexion; (3) hand closing and opening; and (4) elbow extension (Benninger et al., 2011). Before starting the study, patients practiced both motor tasks until they were able to perform them properly. Then, they were abstained from any further practice to minimize learning effects.

Overall motor symptoms assessment was carried out by the Italian validated MDS-UPDRS part III (Antonini et al., 2013).

### Data Analysis

Prior to analyses, all data were tested for normality using the Shapiro-Wilk test. As no violations of the assumption of normality was observed (*p* > 0.05), repeated-measures analyses of variance (rmANOVAs; *p* > 0.05) were carried out.

Changes in the RMT values and in the post/pre-MEP ratios recorded contralaterally to the targeted hemisphere were evaluated by using two-way rmANOVAs with within-subjects factors “Hemisphere” (2 levels: more- and less-affected M1, referring to the targeted hemisphere) and “Condition” (4 levels: baseline, anodal tDCS, cathodal tDCS, sham tDCS).

Three-way rmANOVAs with within-subjects factors “Hemisphere” (2 levels as above), “Side of the body” (2 levels: more- and less-affected body side) and “Condition” (4 levels as above) were carried out to assess changes in the FT rate, in the time to perform the test of upper limb bradykinesia, and in the lateralized MDS-UPDRS scores between the baseline and the post-tDCS sessions. A two-way rmANOVA with within-subjects factors “Hemisphere” (2 levels as above) and “Condition” (4 levels as above) was performed for the total MDS-UPDRS (part III) score.

If the rmANOVAs showed significant differences, Duncan *post hoc* test was used for multiple comparisons. The sphericity assumption was checked by using Mauchly’s test, and the Huynh-Feldt’s correction was adopted, if necessary, for the degrees of freedom. Pearson’s test was used for assessment of correlation between clinical (mean FT rate, time to perform the test of upper limb bradykinesia, total and lateralized MDS-UPDRS scores) and electrophysiological (RMT and post/pre MEP ratios) parameters. Statistical analyses were done with Statistica 7.0 software (StatSoft, Tulsa, OK, USA). For all analyses the level of statistical significance was set at *p* < 0.05.

## Results

The experimental procedures were well tolerated and no adverse effects were reported by any of the participants. Fourteen patients (8M/6F, aged 58 ± 12.1 SD) completed the entire study evaluations. Two patients dropped out due to personal reasons not related with the experimental procedures and were excluded from the statistical analyses (Supplementary Table S1). None of the patients underwent any treatment adjustment throughout the course of the study.

### Electrophysiological Assessment

There were no significant main effects for the RMT as shown by the rmANOVA. The mean RMT values (± SD) recorded in the different sessions were as follow: (1) For the more-affected M1, baseline: 51 ± 6, post-anodal tDCS: 51 ± 6, post-cathodal tDCS: 50 ± 7, post-sham tDCS: 52 ± 6; (2) for the less-affected M1: baseline: 52 ± 7, post-anodal tDCS: 52 ± 8, post-cathodal tDCS: 53 ± 7, post-sham tDCS: 53 ± 7.

When evaluating changes in the post/pre MEP ratios, we observed a significant effect of factor “Hemisphere” *F*_(1,13)_ = 8.74, *p* = 0.011) and a significant interaction between “Hemisphere” and “Condition” *F*_(3,33)_ = 5.85, *p* = 0.004; Figure 2). The *post hoc* analysis showed that: (1) the post/pre MEP ratio significantly increased after anodal tDCS of the more-affected M1 in comparison with both the baseline (*p* = 0.004) and the sham (*p* = 0.018) condition; (2) the post/pre MEP ratio significantly decreased after cathodal tDCS of the less-affected M1 in comparison with both the baseline (*p* = 0.001) and the sham (*p* = 0.011) condition. The mean values (± SD) of the stimulation intensity used to obtain a 1 mA MEP amplitude in the different sessions were as follows: (1) for the more-affected M1, baseline: 66 ± 9, anodal tDCS: 68 ± 9, cathodal tDCS: 68 ± 8, sham tDCS: 69 ± 8; (2) for the less-affected M1: baseline: 65 ± 12, anodal tDCS: 66 ± 12, cathodal tDCS: 66 ± 10, sham tDCS: 68 ± 8.

### Finger Tapping Test

**Figure 3:**
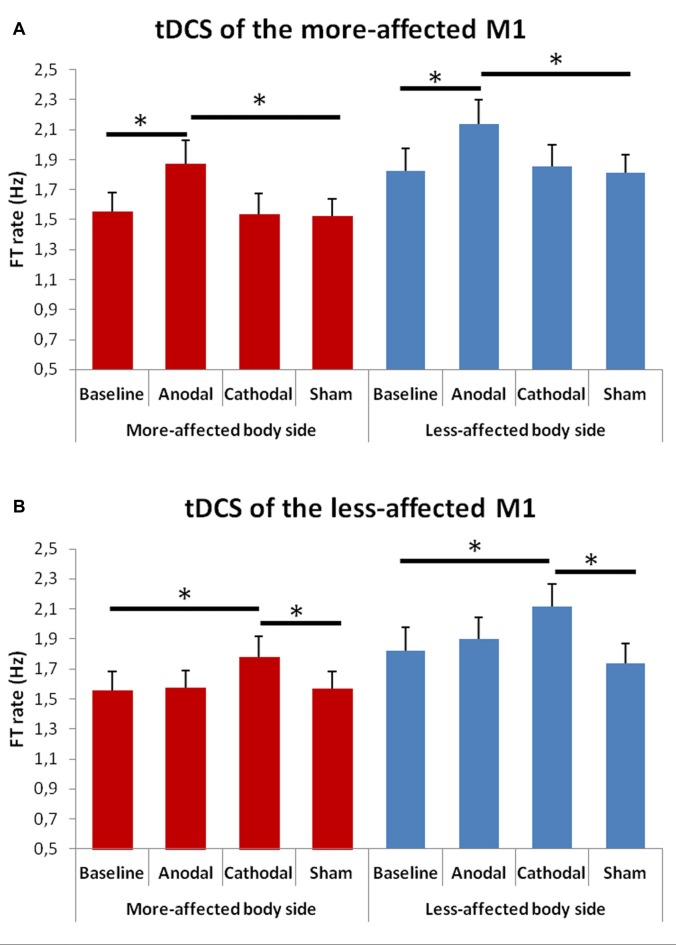
Changes in total mean FT rate (50 cycles) in the more- **(A)** and in the less-affected **(B)** side of the body between the baseline and the different tDCS sessions. Error bars indicate standard error of means (SEM). Horizontal bars indicate significant differences (*p < 0.05).

When comparing the FT rate between the baseline and the tDCS sessions (Figure 3) the rmANOVA showed a significant effect of factors “Condition” *F*_(4,48)_ = 3.39, *p* = 0.001) and “Side of the body” *F*_(1,13)_ = 19.63, *p* = 0.001), and a significant interaction between “Hemisphere” and “Condition” *F*_(3,23)_ = 11.02, *p* = 0.001).

At the *post hoc* analysis, we observed that, after both anodal tDCS of the more-affected M1 and cathodal tDCS of the less-affected hemisphere, the FT rate significantly increased in the more- and in the less-affected hand as compared to both the baseline and the sham condition (*p* < 0.001 for each comparison).

### Upper Limb Bradykinesia

**Figure 4:**
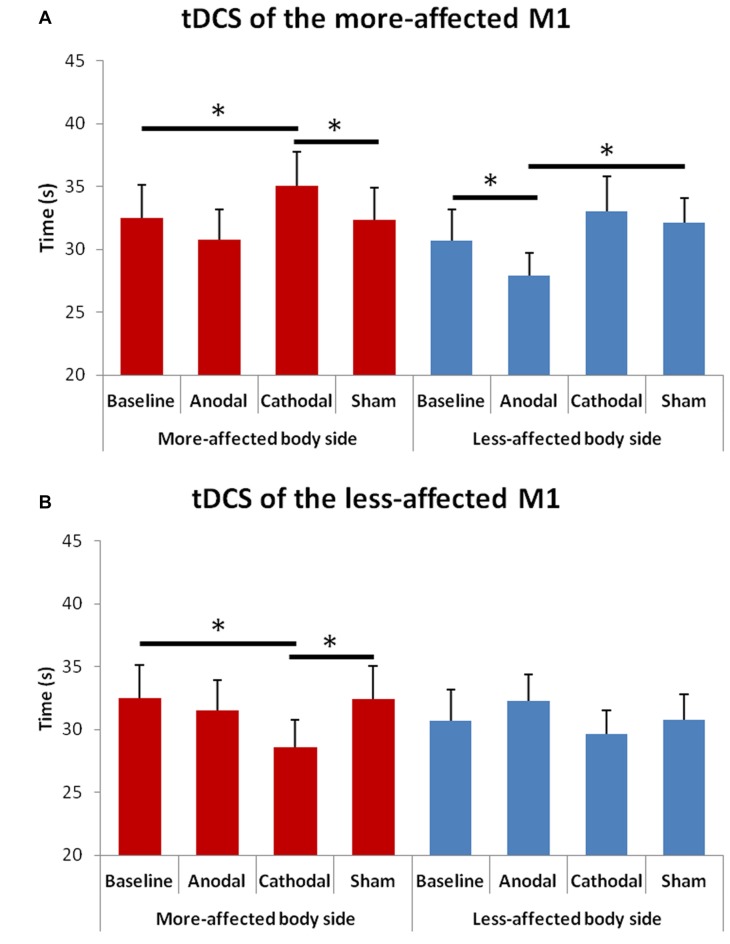
Changes in upper limb bradykinesia in the more- and in the less-affected side of the body after tDCS of the more- **(A)** or the less-affected **(B)** M1. Error bars indicate standard error of means (SEM). Horizontal bars indicate significant differences (*p < 0.05).

The rmANOVA for bradykinesia of the upper limbs (Figure 4) showed a significant effect of factor “Hemisphere” *F*_(1,13)_ = 8.13, *p* = 0.014) and significant interactions between “Hemisphere” and “Condition” *F*_(3,23)_ = 11.02, *p* = 0.001) and between “Hemisphere” and “Side of the body” *F*_(1,13)_ = 8.13, *p* = 0.015).

At the *post hoc* analysis, we observed that: (1) in the more-affected hand, the time to perform the test significantly reduced after cathodal tDCS of the less-affected M1 as compared to both the baseline (*p* = 0.005) and the sham (*p* = 0.006) condition; (2) in the more-affected hand, the time to perform the test significantly increased after cathodal tDCS of the more-affected M1as compared to both the baseline (*p* = 0.035) and the sham (*p* = 0.037) condition; and (3) in the less-affected hand, the time to perform the test significantly reduced after anodal tDCS of the more-affected M1 as compared to both the baseline (*p* = 0.002) and the sham (*p* = 0.026) condition.

### Total and Lateralized MDS-UPDRS (Part III) Scores

**Figure 5:**
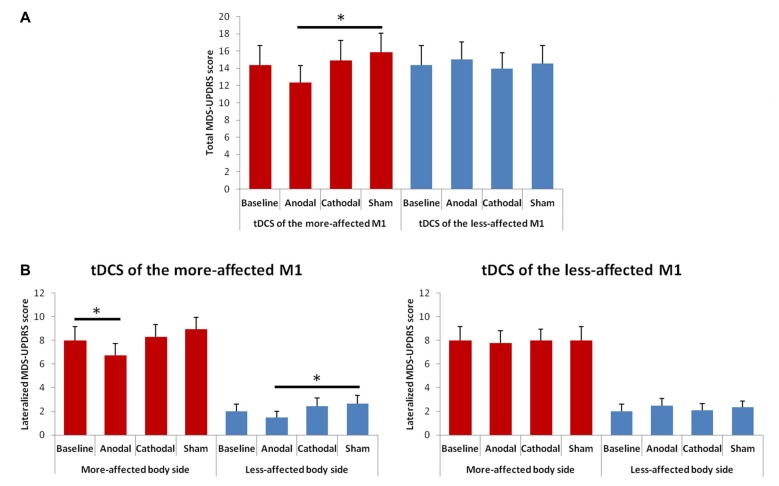
Changes in total **(A)** and lateralized **(B)** MDS-UPDRS (part III) scores between the baseline and the different tDCS sessions. Error bars indicate standard error of means (SEM). Horizontal bars indicate significant differences (*p < 0.05).

The rmANOVA for the total MDS-UPDRS (part III) score showed a significant interaction between “Hemisphere” and “Condition” *F*_(3,39)_ = 3.52, *p* = 0.023; Figure 5). At the *post hoc*, we observed a significant score reduction after anodal tDCS of the more-affected M1 as compared to the sham session (*p* = 0.023); a trend towards significance between the post-anodal and the baseline session (*p* = 0.061) was also shown.

The rmANOVA for changes in the lateralized MDS-UPDRS scores showed a significant effect of factors “Side of the body” *F*_(1,13)_ = 110.48; *p* = 0.001) and “Condition” *F*_(3,39)_ = 2.96, *p* = 0.044), and significant interaction between “Hemisphere” and “Condition” *F*_(3,39)_ = 3.00, *p* = 0.041; Figure 5). At the *post hoc* analysis, we observed a significant score reduction in the more-affected side of the body after anodal tDCS as compared to the baseline (*p* = 0.016) and a trend towards significance between the post-anodal and the sham session (*p* = 0.060). In the less-affected side of the body, we observed only a significant difference between the anodal and the sham session (*p* = 0.036).

### Correlations between Clinical and Electrophysiological Parameters

**Figure 6:**
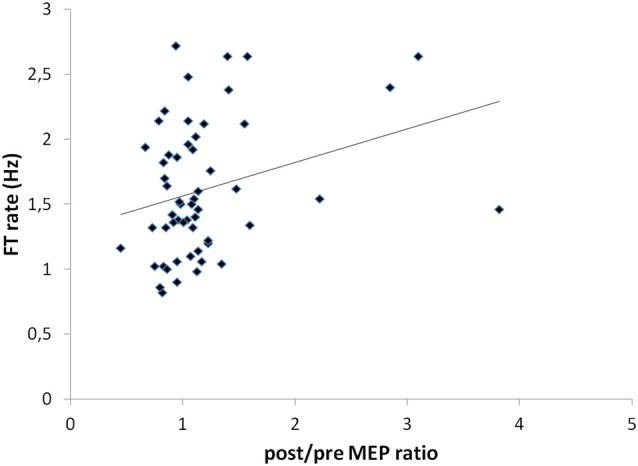
Correlation between post/pre MEP ratios and FT rate values recorded in the more-affected hand (*r* = 0.292; *p* = 0.03).

A significant correlation was shown between the post/pre MEP ratios and the FT rate recorded in the more-affected hand (*r* = 0.292; *p* = 0.03; Figure 6). No other significant correlations were found between other clinical and electrophysiological parameters.

## Discussion

In the present study, we found that in PD patients with asymmetric motor symptoms, the neurophysiological and clinical effects of tDCS applied to the M1 strictly depend on the type of current used (anodal vs. cathodal) and the side of stimulation (more- vs. less-affected M1). We showed that: (1) different cortical plastic changes were induced by tDCS in the M1 of the two hemispheres in patients with asymmetric PD; (2) only anodal tDCS of the more-affected M1 and cathodal tDCS of the less-affected hemisphere induced a clinical improvement.

### Electrophysiological Assessment

Several previous works have reported abnormal cortical plasticity responses to different NIBS techniques in PD patients both on- and off-medication (Fregni et al., 2006; Stephani et al., 2011; Suppa et al., 2011; Kishore et al., 2012; Kojovic et al., 2012; Zamir et al., 2012; Udupa and Chen, 2013). However, very few studies have examined both hemispheres (Kishore et al., 2012; Kojovic et al., 2012). Our findings on changes in post/pre MEP ratios showed that very different polarity-dependent changes in cortical excitability may be induced by tDCS when it is applied to the two hemispheres of PD patients on-medication. In particular, we observed that anodal tDCS induced a significant MEP potentiation only when applied to the more-affected M1, while, conversely, cathodal tDCS resulted in MEP inhibition only when the less-affected hemisphere was targeted.

The present results could help to explain previous conflicting findings of either absent LTP-like responses (Stephani et al., 2011; Suppa et al., 2011; Kishore et al., 2012), or reduced (Fregni et al., 2006) or absent (Kishore et al., 2012) LTD-like plasticity, or even normal synaptic plasticity (Zamir et al., 2012) in PD. Indeed one relevant reason for such differences might be that, along with methodological differences and clinical heterogeneity, care has not always been paid to which hemisphere is being studied.

Mechanisms underlying the observed changes in motor cortical excitability remain speculative. In line with suggestion that cortical metaplasticity may be involved in the pathophysiology of several neuropsychiatric disorders including PD (Karabanov et al., 2015), metaplasticity changes could be invoked to explain our results. Cortical metaplasticity refers to a variety of processes that regulate the plastic state of the neural networks also including sliding thresholds for long-term potentiation (LTP) and long-term depression (LTD; Bienenstock et al., 1982). In this view, the lack of inhibitory effect of cathodal tDCS in the more-affected hemisphere could be interpreted as consequence of an increase in the threshold for eliciting LTD, whilst the lack of facilitatory effect of anodal tDCS in the less-affected M1 could be expression of an increase in the threshold for LTP induction. If so, the observed modifications in cortical plasticity could represent, at least theoretically, a process that takes place in the central nervous system in the attempt to compensate for the interhemispheric functional imbalance. Other potential mechanisms, however, should be taken into account to explain the observed results. The first includes modifications in the degree of functional asymmetry at the cortical level, that could have been consequence of changes in mutual transcallosal inhibition induced by the stimulation of one hemisphere. Future studies investigating changes in excitability of the M1 contralateral to that stimulated and changes in transcallosal inhibition are needed to address this issue. The second mechanism involves trans-synaptic modulation of cortico-striatal and thalamo-cortical circuits, according to the evidence that tDCS can modulate functional connectivity of the cortico-subcortical circuits (Polanía et al., 2012).

### Clinical Evaluation

It has been hypothesized that in PD, a functional asymmetry in M1 excitability could contribute to the motor impairment by leading to an unbalanced interhemispheric inhibition unfavoring the more-affected hemisphere (Li et al., 2007; Spagnolo et al., 2013; Verheyden et al., 2013). The present results seem to support this suggestion showing that both anodal tDCS of the more-affected M1, which was proven to enhance motor cortical excitability, and cathodal tDCS of the contralateral less-affected M1, which resulted in a decrease in motor cortical excitability, improved motor symptoms in PD. It is also noteworthy that both of these two tDCS montages were shown capable to induce beneficial effects on the motor performance not only on the contralateral side of the body, but also ipsilaterally. This could be due to a rebalancing effect on interhemispheric communication induced by unilateral tDCS, which in turn could improve motor function also in the contralateral hemisphere, according to experimental findings that lateralization of movement depends on complex interhemispheric communication between cortical and subcortical regions (Cincotta and Ziemann, 2008; Beaulé et al., 2012; Welniarz et al., 2015). In particular, our results could be interpreted in the light of the hypothesis that while cortical hyperexcitability could represent a compensatory strategy to compensate for the reduced cortical preactivation level in the more-affected hemisphere, in the less-affected one it could be maladaptive. Indeed, anodal tDCS of the more-affected M1 could result not only in increased ipsilateral cortical activity, as suggested by increase in MEP amplitudes, but also in enhanced transcallosal inhibition to the contralateral less-affected M1. Conversely, cathodal tDCS of the less-affected M1 could not only reduce maladaptive ipsilateral cortical hyperexcitability, as suggested by the amplitude reduction of MEP, but contextually it could decrease transcallosal inhibition to the more-affected hemisphere. However this interpretation remains speculative and future research is needed to evaluate which changes in transcallosal inhibition may be induced by unilateral cathodal or anodal tDCS of the two motor cortices and what the impact might be for motor function of the two sides of the body in PD patients.

In this study, a significant positive correlation was observed only between changes in the FT rate in the more-affected hand and changes in the MEP values recorded from the same side. This suggests that the clinical improvement induced by tDCS was not strictly related to shifts in the M1 output, but more complex, even multiple synergic mechanisms were probably involved, including along with a possible effect on transcallosal interactions between the two hemispheres also indirect modulatory effects on the basal ganglia function (Polanía et al., 2012).

Future investigations are needed to clarify to which extent the dopaminergic treatment could have influenced our results. Here we evaluated only patients while on treatment as our main aim was to investigate the therapeutic potential of tDCS in addition to the pharmacological therapy. However, though an “on” vs. “off” comparison could be of little interest in the daily clinical setting of the patients, it could have been of considerable interest when evaluating a theoretical model. In this regard it may be of interest the recent finding by Costa-Ribeiro et al. (2016b) showing that anodal tDCS applied to the frontal motor cortex can prolong the positive effects of cued gait training in PD patients both in on and off medication state. The authors also evaluated changes in cortical excitability without finding any tDCS-induced change on MEP amplitudes in patients both on and off treatment. Such a difference compared to our results could be due to various factors including different electrode montages, and concomitant use of tDCS and motor training.

It is to note that partly different response patterns were observed by using the FT test and the test of upper limb bradykinesia. One possible explanation to such difference is that, as compared to the FT movements, the planning of more complex movements of the upper limbs requires involvement of higher cortical planning areas, such as the supplementary motor area and the premotor cortex of the two hemispheres. If so, it is possible that different effects of tDCS on upper limb bradykinesia could have been observed by targeting other cortical areas. On that account, however, it is to note that the large electrodes normally used for tDCS are ill-suited for focal targeting, and thus we cannot rule out that some of the observed tDCS-induced effects were due to an influence on activity of brain areas located in close proximity to the M1.

It is also noteworthy that a worsening of bradykinesia of the more-affected upper limb was noticed after cathodal tDCS of the corresponding more-affected M1. This finding, in agreement with previous reports of possible decline in motor performances after tDCS in PD (Fregni et al., 2006), strengthen the importance to carefully choose the best cortical target and stimulation parameters to treat a given patient. Another aspect to be considered regards the emerging information about the role of age-associated brain reorganization in the clinical response to different tDCS protocols applied with the aim to improve functional impairment. Indeed, though encouraging results have been reported in older adults, evidence has been also provided that different, even opposite effects may be observed when different tDCS montages are applied to improve motor and cognitive function in elderly subjects as compared to young individuals (Dumel et al., 2016; Perceval et al., 2016; Fujiyama et al., 2017). Thus in the future, not only disease-related brain abnormalities but also age-related brain plastic changes will have to be taken into account when planning a tDCS treatment in patients with PD.

As regards the MDS-UPDRS scores, we observed a significant reduction in the lateralized MDS-UPDRS scores, as compared to the baseline, only in the more-affected side of the body after anodal tDCS of the more-affected M1. Similar considerations as those for bradykinesia should be made, considering that the MDS-UPDRS evaluates patients motor abilities involving different neural pathways. In addition, it should be considered that the MDS-UPDRS could be less sensitive in detecting subtle differences in motor performances with respect to the other motor tasks used, especially in the less-affected side of the body where lower scores were recorded.

## Limitations and Conclusions

One of the main limitation of this study is probably that we cannot rule out that the observed clinical changes between sessions were, at least partly, confounded by day-to-day clinical fluctuations not related to the brain stimulation. The use of a pre/post tDCS design for the clinical assessment could have limited this potential confounding factor. However, this paradigm was not used because of the need to perform the clinical assessment always at the same time interval from the last drug intake, in the baseline as well as after each tDCS intervention. It is nevertheless important to point out that all subjects enrolled presented a fairly stable motor impairment, as assessed during a 2-month run-in period, and none of the patients was in the advanced stage of the disease or presented motor fluctuations.

Other potential limitations refer to the absence of a control group with age-matched subjects, and to the sham method applied. On this latter point, though we used a well-standardized procedure (Nitsche et al., 2008), some authors have raised the possibility that, at least in some patients, a complete blinding could not be achieved with a 2 mA stimulation intensity (O’Connell et al., 2012). It is noteworthy, in this regard, that we checked for blindness by asking all patients, at the end of the study, to disclose any significant difference between the different procedures performed. None of the participants was able to distinguish between real and sham tDCS. This finding, unlike that obtained in healthy young subjects, could be attributed to a poor attitude of the patients to get an in-depth understanding of the study protocol, at least as far as the existence of a sham condition. One might suppose that this could be due to peculiar PD-related and/or age-related cognitive processes, though targeted studies would be needed to address this issue.

Some other potential sources of bias refer to the fact that we did not record the FT movements by using a surface electromyogram (EMG) or an accelerometer, and to the relatively low number of MEPs that were computed. In this regard, although we used a previously described procedure (Fregni et al., 2006), a recent work by Chang et al. (2016) suggests that to have a reliable measure of M1 excitability it would be recommended to record at least 20 MEPs per block. Moreover, the interpretation of MEP changes induced by the different tDCS interventions should take into account the possibility that, especially in patients with resting tremor, tDCS also induced changes in the background muscle activation which in turn could affect MEP amplitudes. Though an analysis of the pre-trigger EMG activity was not carried out, some clinical clues from evaluation of unilateral MDS-UPDRS sub-item scores for resting tremor of the upper limbs (Supplementary Table S2) seem to suggest that MEP changes were mainly due to modification in cortical/transcallosal excitability. For instance anodal tDCS of the more-affected hemisphere determined a reduction in the mean MDS-UPDRS sub-item score for resting tremor of the contralateral hand along with an increase in MEP amplitude, and it also determined a clinical improvement ipsilaterally.

Another aspect that deserves to be discussed refers findings by some authors that in PD anodal tDCS can improve motor performances such as gait and balance only when combined with physical therapy (Kaski et al., 2014; Costa-Ribeiro et al., 2016a), likely thanks to the ability of anodal tDCS to enhance implicit motor learning (Nitsche et al., 2003b). On that account, future investigations are needed to assess whether the effects of the different tDCS montages on the upper limb motor function could be different or even more pronounced when applied in conjunction with a rehabilitation training program.

In conclusion, our findings strengthen the notion that tDCS has a therapeutic potential in PD, but also point out to the need to carefully personalize patients’ treatment settings. Indeed, here we show that in PD patients with asymmetric motor symptoms, maximum benefit on motor functions by tDCS could be gained by choosing the hemisphere to stimulate based on the most affected body side at clinical examination.

The present results should be considered preliminary as they are based on a relatively small sample size, and they should be confirmed in larger, prospective trials also evaluating efficacy and safety of multiple tDCS sessions repeated over time.

## Author Contributions

GC: drafting/revising the manuscript, study concept and design, data collection, analysis and interpretation of the data. FV: revising the manuscript, study concept and design, data collection and interpretation of the data. MT and RD: revising the manuscript, data collection and interpretation of the data. EA and GS: revising the manuscript, study concept and design and interpretation of the data. BF, MDA and FB: revising the manuscript, study concept and design, analysis and interpretation of the data.

## Conflict of Interest Statement

The authors declare that the research was conducted in the absence of any commercial or financial relationships that could be construed as a potential conflict of interest.
