# Supplementary material for: Effects of More-Affected vs. Less-Affected Motor Cortex tDCS in Parkinson’s Disease
Source: Front Hum Neurosci. 2017 Jun 12;11:309. doi: 10.3389/fnhum.2017.00309 (PMC5466958; doi:10.3389/fnhum.2017.00309)
Supplement: Supplementary file 1 [file Table_1.docx]

***TABLE 1:*** *Clinical characteristics of the Parkinson’ disease (PD) patients enrolled.*

| **Subject** | **Age, gender**  **(handedness)** | **More-affected**  **side** | **H&Y** | **Motor**  **MDS-UPDRS** | **More-affected side**  **MDS-UPDRS** | **Less-affected side**  **MDS-UPDRS** | **PD medication** |
| --- | --- | --- | --- | --- | --- | --- | --- |
| 1 | 54 M (Rh) | R | 1 | 10 | 4 | 0 | LD |
| 2 | 76 F (Rh) | R | 2,5 | 18 | 10 | 2 | LD, DA |
| 3 | 41 F (Lh) | L | 1 | 5 | 3 | 0 | MAO |
| 4 | 45 M (Rh) | L | 1 | 4 | 2 | 0 | LD, MAO,DA |
| 5 | 61 F (Rh) | R | 1 | 6 | 3 | 0 | DA, MAO |
| 6 | 69 F (Rh) | R | 2 | 13 | 7 | 2 | LD, MAO,DA |
| 7 | 66 M(Rh) | R | 2 | 17 | 9 | 3 | LD, A |
| 8 | 44 F (Rh) | R | 2 | 14 | 9 | 2 | LD |
| 9 | 47 M (Rh) | R | 1 | 7 | 3 | 0 | DA |
| 10 | 64 M (Rh) | R | 2,5 | 19 | 9 | 3 | LD, MAO,DA |
| 11 | 52 F (Rh) | R | 1 | 3 | 3 | 0 | LD, MAO,DA |
| 12 | 66 F (Rh) | R | 2 | 12 | 9 | 1 | DA, MAO |
| 13 | 76 F (Rh) | R | 2 | 22 | 11 | 5 | DA, LD |
| 14 | 65 M(Rh) | R | 2 | 33 | 17 | 7 | DA,LD |
| 15 | 57 M (Rh) | R | 2 | 24 | 14 | 4 | DA, MAO, LD |
| 16 | 45 M (Rh) | L | 1 | 6 | 5 | 0 | DA, MAO |

Rh, Lh denote handedness for each subject; R, L indicated right or left side of worse PD symptoms.

H&Y, Hoen &Yahr score; MDS-UPDRS, MDS-Unified Parkinson’s Disease Rating Scale in ON state; LD, levodopa; DA, dopamine agonist; MAO, monoamine oxidase inhibitor; A, amantadine.

Subjects 11 and 12 dropped out the study.
