# Supplementary material for: Effects of More-Affected vs. Less-Affected Motor Cortex tDCS in Parkinson’s Disease
Source: Front Hum Neurosci. 2017 Jun 12;11:309. doi: 10.3389/fnhum.2017.00309 (PMC5466958; doi:10.3389/fnhum.2017.00309)
Supplement: Supplementary file 2 [file Table_2.docx]

**Table 2.** Unilateral sub-item scores (mean ± SD) from the Italian validated Movement Disorder Society revision of the Unified Parkinson’s Disease Rating Scale (MDS-UPDRS) for resting tremor of the upper limbs in baseline and after different tDCS montages. Clinical assessment was performed in patients on medication.

|  | ***More-affected body side*** | ***Less-affected body side*** |
| --- | --- | --- |
| **Baseline** | 0,86 ± 0,95 | 0,07 ± 0,26 |
| ***More-affected primary motor cortex (M1)*** |  |  |
| **Anodal tDCS** | 0,72 ± 0,99 | 0,00 ± 0,00 |
| **Cathodal tDCS** | 1,00 ± 0,88 | 0,07 ± 0,26 |
| **Sham tDCS** | 1,07 ± 0,92 | 0,07 ± 0,26 |
| ***Less-affected primary motor cortex (M1)*** |  |  |
| **Anodal tDCS** | 1,07 ± 0,92 | 0,14 ± 0,35 |
| **Cathodal tDCS** | 1,07 ± 0,92 | 0,00 ± 0,00 |
| **Sham tDCS** | 1,00 ± 0,96 | 0,07 ± 0,26 |
